# Surface Modifications of Polyetheretherketone (PEEK): Results from the Literature and Special Studies of Copper-Coated Films

**DOI:** 10.3390/polym14224797

**Published:** 2022-11-08

**Authors:** Helmut Münstedt, Joern Grossmann

**Affiliations:** 1 Institute of Polymer Materials, Friedrich-Alexander-Universität Erlangen-Nürnberg, Martensstr. 7, 91058 Erlangen, Germany; 2 SEMIKRON Elektronik, 90431 Nürnberg, Germany

**Keywords:** polyetheretherketone, copper layers, surface treatment, surface analysis, bonding properties

## Abstract

For some challenging applications of the high-performance thermoplastic polyetheretherketone (PEEK) in engineering or medical fields, the bonding to other materials is important. In these cases, modifications of the inert polymer surface are often necessary. Results from the literature with respect to sandblasting, corona discharge, plasma treatment, excimer-laser irradiation, and chromium-sulfuric-acid etching are described and discussed. Our own detailed studies of these methods under well-defined experimental conditions are reported, which make their comparability possible and support the assessment for certain applications. PEEK films coated with copper are of special interest due to their potential for flexible electronics. Copper foils glued by an epoxy resin and copper layers from physical vapor deposition are compared with respect to their mechanical adhesion. Surface properties were characterized with respect to roughness, contact angle, and oxygen-to-carbon (O/C) ratio. The latter has been found to be the most decisive parameter for good adhesion. It was shown that an enhancement of the O/C ratio can be achieved in several ways. The advantages and disadvantages of the methods applied are discussed under various aspects of applications.

## 1. Introduction

Polyetheretherketone (PEEK) is a high-temperature-resistant engineering polymer used for special applications. It has a glass transition temperature of T_g_ = 143 °C, a melting temperature of T_m_ = 340 °C and a continuous use temperature of T_CU_ = 260 °C, according to UL 746B [1]. Besides its good mechanical properties, PEEK is relatively inert with respect to a lot of substances and, thus, environmental influences. PEEK is a thermoplastic polymer and can be processed by well-known operations such as extrusion, injection molding, thermoforming or additive manufacturing. This convenient processability has led to a great number of applications with special challenging requirements in the automotive and aerospace industry as well as in fields of electronics, energy, and medical engineering. Special PEEK grades have gained access to dental and orthopedic implants [2,3]. In a recent literature review actual and future applications of PEEK in technical and medical fields are summarized and some fundamental results on surface modifications of PEEK are addressed [4].

For some applications, parts from PEEK are joined to each other or combined with other materials. In such cases, adhesives are used or layers are directly deposited. Their adhesion depends on the interaction with PEEK molecules at the surface of an item. Increasing the low surface energy of PEEK is a precondition for a good bonding performance. That can be reached, for example, by the introduction of polar groups to the polymer substrate. In case of PEEK, this aim is particularly challenging because of its chemical inertness. Various chemical and physical processes have been applied over the years and have been reported in the literature. However, often the results are not comparable because the materials were not the same or the pretreatments were not described in enough detail. This paper tries to close this gap by comparing published results and describes our own experiments at definite conditions, from which the efficiencies of the various methods can be assessed. The studies intend to provide a basis to identify the most convenient way of modifying the surface of PEEK for a special application. Furthermore, detailed analyses of the processes and characterizations of the surfaces are reported, which support a deeper understanding.

## 2. Materials and Characterization Methods

### 2.1. Materials

**Figure 1:**
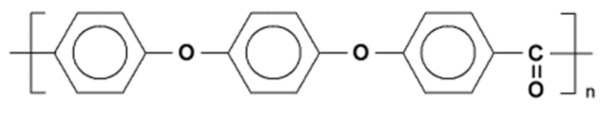
Chemical structure of PEEK.

Victrex PEEK^R^ [1], the chemical structure of which is shown in Figure 1 was used in all the experiments. For measuring the adhesive performance of glued part by lap strength tests, samples were injection molded at a mass temperature of 390 °C and a mold temperature of 180 °C. The substrates for flexible copper-coated composites were commercial PEEK films with thicknesses of 50 μm.

For the lamination of copper foil on PEEK, a heat curing epoxy adhesive with a continuous use temperature of 280 °C was applied. Weiss [5] studied the efficiency of various adhesives with respect to the adhesion strength of copper foils on flexible PEEK substrates. Significant differences in preparation and properties of the composites were found, but the tendency of the pretreatment effects was the same. As the effect of surface modifications on PEEK/copper laminates is the main topic of this paper, the restriction to one adhesive is seen to offer a good enough generality for studying the efficiency of various surface treatments. The two-component epoxy adhesive Duralco 4460 of Polytec was used for all the experiments performed.

The PEEK films were laminated with a commercial copper foil of 18 μm thickness. Alternatively, a direct metallization with copper was carried out by physical vapor deposition (PVD) using a cathodic sputtering unit described in [5].

### 2.2. Characterization Methods

An application-related characterization of glued composites is mechanical tests of the adhesion strengths. For polymer/polymer joints, the measurement of the lap strength is a usual method, and for polymer/metal laminates, the peel strength is an appropriate quantity. The adhesion of the thin PVD layers of around 3 μm was studied by using the forehead pull test.

To obtain a deeper insight into the bonding mechanisms, characterizations of the treated surfaces are important. An obvious quantity is the surface roughness. Surface energies assessable from the determination of the contact angles provide information on the spreading behavior of a liquid on a solid. The chemical composition of a surface and the existence of polar groups, in particular, may be helpful in understanding interactions between substrate and adhesive.

#### 2.2.1. Lap Shear Strength

The measurement of the lap strength is based on the tensile shear test according to the norm DIN 53283 [6] using tensile bars according to DIN 53455 [7]. Special mechanical devices are applied to obtain a reproducible distribution of the adhesive in the overlapping region. The samples are tested in a tensile testing machine at a deformation rate of 5 mm/min. The lap strength is calculated as the maximum force related to the overlapping area. This quantity can be determined with some uncertainties only, due to irregularities of the adhesive distributions at the rims of the overlap.

#### 2.2.2. Peel Strength

**Figure 2:**
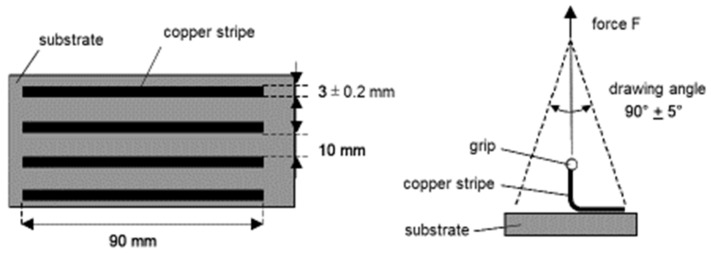
Essentials of the peel test. Geometry of the test samples (**left**) and sketch of the test procedure (**right**).

For the adhesion of foils or metal coatings, the peel test according to DIN EN 60249 [8] is used. The geometry of the test samples and the principle of the test procedure are shown in Figure 2. Metal stripes can conveniently be obtained by a photoresist etching procedure as described in [5], for example. They are pulled at an angle close to 90° in a tensile testing machine at a haul-off speed of 50 mm/min over a length of at least 25 mm. The drawing force is recorded as a function of sample length and, thus, can be conveniently averaged. The peel strength is obtained by relating the force to the sample width. The application of this method is restricted to coatings of tear strengths higher than the adhesion forces and, therefore, coating thicknesses may not fall below distinct values.

A way to apply the peel test to thin metal layers as typically generated by physical vapor deposition (PVD), for example, is reinforcement in a galvanic process. However, such a procedure may influence the interactions between coating and substrate materials and thus is not used in this paper.

#### 2.2.3. Forehead Pull Test

**Figure 3:**
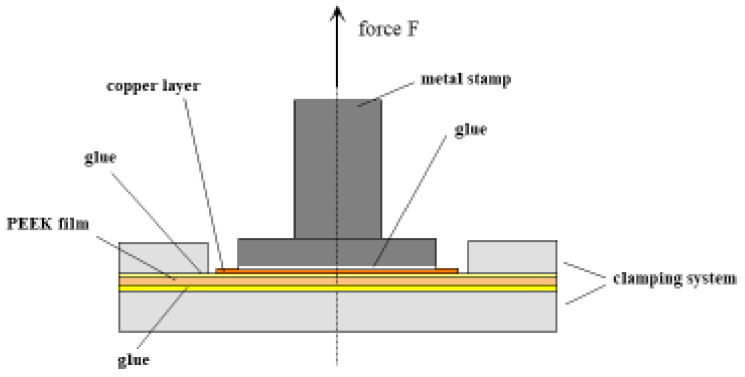
Forehead pull test according to DIN EN ISO 4624.

For the study of the adhesion of thin layers on substrates, a pull-off test according to DIN EN ISO 4624 [9] was developed. It is sketched in Figure 3 for PEEK/copper laminates. The PEEK film/copper foil laminate or the PEEK film directly metallized by PVD is glued to a mechanically stable frame and additionally fastened by clamps. A metal stamp is fixed to the copper layer by an adhesive that has to be stronger than the adhesion between the layer and PEEK. The device is mounted to a tensile testing machine and the stamp is drawn up to the failure of the composite at a rate of 10 mm/min. The maximum force related to the surface area of the stamp is used as the characteristic quantity for the adhesion properties of the composite. It is obvious that this value stands for the weakest point of the composite and does not reflect an average as in the case of peel strength. Furthermore, the differentiation of failure properties becomes rather inaccurate when the adhesion between layer and substrate approaches that between layer and metal stamp, given by the adhesive used.

#### 2.2.4. Types of Failure

Different types of failure may occur, and the visual description of their features may become interesting for an optimization of composites. A detachment of the adhesive from the glued parts or a separation of coatings from a substrate is called adhesive failure, and the breakage of one of the joined partners is cohesive failure. Furthermore, a cohesive failure of the adhesive itself may be observed. In some cases, different features are superimposed on each other.

#### 2.2.5. Measurement of Roughness

The surface roughness is a parameter that may influence adhesion. It can be measured by commercial tactile devices. Systems that work without any mechanical contact are based on laser optics (see, for example, DIN EN ISO 25 178 [10]). Commercial instruments provide resolutions of 1 µm in horizontal directions and about 0.01 µm perpendicular to the surface. A still higher resolution can be obtained by scanning electron microscopy, but this method needs some special experimental efforts.

#### 2.2.6. Measurement of Contact Angle

The contact angle provides information on the surface energy between a liquid and a solid and can be used to qualitatively assess the spreading behavior of an adhesive on the surfaces of parts to be joined. A small contact angle indicates a good wetting of the surface of a solid by a liquid. Commercial instruments are available to determine contact angles and, thus, they are routinely used for surface characterizations. An introduction to the relationship between contact angle and surface energy and the procedures to determine them are described, for example, in [11]. An assessment of the various experimental methods can be found in [12]. At this point it should be mentioned that roughness may influence wettability and, consequently, the surface angle to some extent.

#### 2.2.7. Electron Spectroscopy

The electron spectroscopy for chemical analysis (ESCA), alternatively called X-ray photoelectron spectroscopy (XPS), is applied to obtain information about the chemical composition of a surface, which supports the basic understanding of treatments for improving adhesion properties. The technique needs sophisticated equipment and a lot of experimental experience. Detailed descriptions of the method can be found in special textbooks (e.g., [13]). From overview spectra, data with respect to the chemical composition of a surface can be obtained, and from the deconvolution of special spectral lines, conclusions with respect to the special binding states of an element can be drawn.

## 3. Various Treatments and Their Effects on Surface Properties and Adhesive Bonding

### 3.1. Sandblasting

#### 3.1.1. Results from the Literature

The most obvious intention to increase adhesion is the enlargement of the interacting surface areas. A simple method is roughening, which can be effectively achieved for polymeric materials by blasting with fine hard particles. Some studies on PEEK can be found in the literature, but it is difficult to draw convincing conclusions from them. In [14], the topography and wettability of PEEK samples with different surface roughnesses obtained by blasting with aluminum oxide particles of various sizes were evaluated. No adhesive properties were reported and, furthermore, the contact angle measurements are somewhat confusing. In another paper [15], PEEK was blasted with alumina powder and chemically treated before being connected to a resin cement used in dentistry. The aim of the work was to improve its bond strength to PEEK. The best adhesion was found for a combination of roughening and chemical etching, but neither data on surface characterization nor on the effect of abrasion alone were provided.

Grit blasting with alumina grinding media was performed as one of the treatments within an extensive study on adhesive bonding of PEEK [16]. The adhesion efficiency was determined by measuring the lap shear strength of the pretreated samples. It was shown that abrasion with a silicon carbide paper alone did not improve the adhesion remarkably, but that a subsequent bombardment with the alumina particles resulted in an increase of bond strength, which was found to be lower, however, than that achieved by plasma etching. Characterizations of corresponding surface modifications were not provided.

Results on the surface treatment of PEEK with two different alumina particles are reported in [17]. Air abrasion of plates of a thickness of 2 mm was performed with 50 μm alumina particles and 110 μm silicate-coated alumina particles. The roughness was measured and the surface tension characterized by contact angle measurements. The effectiveness of five adhesives was studied in a special shearing test. The roughness as presented in the paper increased from 0.25 μm for the untreated sample to 2 μm for the blasted samples. The contact angles of the alumina-abraded PEEK were around 20° lower than that of the untreated material and did not differ between the particles of various sizes and coatings. The bonding strength was distinctly enhanced by the previous abrasion. A significant difference within the experimental scatter was neither found between the two blasting media nor the five adhesives used. It is interesting to mention that with respect to the bond strength, the mechanical abrasion appeared to be more effective than etching with 95 % sulfuric acid, which correlates, however, with a comparably lower decay of the contact angle and a smaller increase in roughness.

The effect of an abrasion treatment on adhesion properties of PEEK is briefly addressed in a paper dealing with plasma activation [18]. PEEK was blasted using commercial alumina particles coated with silicate, and it was shown that the lap shear strength of the untreated material could be remarkably enhanced, comparable to the effect of corona treatment and etching with chromium sulfuric acid, but it was found to be less effective than plasma treatments. Surface characterizations such as roughness and wettability are not available and, thus, a deeper insight into the mechanism underlying the improvement of adhesion cannot be obtained from this paper.

#### 3.1.2. Detailed Studies on PEEK

The literature on surface roughening of PEEK is scarce, and comprehensive characterizations of the treated samples are often missing, as the previous section demonstrates. Thus, we performed our own experiments, the results of which are reported in the following sections.

##### Tensile Bars

Tensile bars from Victrex PEEK 450 G were injection molded according to [7] and overlap joints were manufactured using an epoxy resin. The lap strength was determined following [6]. A previous roughening of the glued surfaces was obtained using 600-SiC- sandpaper or by blasting with glass particles of diameters between 100 and 200 μm. The mean roughness of the surfaces characterized by a contact-free laser-optical device increased from 0.12 μm for the initial sample to 0.79 μm for the sandblasted and 2.57 μm for sandpaper-treated specimens [19]. The lap shear strength obtained with a two-component epoxy resin increased from 0.7 MPa to 2 MPa by roughening. Within the accuracy of the measurement, a difference between the two surface treatments was not found. All the samples showed an adhesive failure that means the epoxy resin completely separated from the polymer surface.

##### Films

The effect of surface roughening on the strength of glued PEEK joints depends on many variables, such as the joint partners, for example. This becomes obvious from the following experiment. A 50 μm PEEK film was blasted with the same glass beads as the tensile bars above. Subsequently, an 18 μm copper foil with a special galvanic surface treatment to increase its roughness (Schlenk AG, Roth, Germany) was glued to the untreated and the roughened PEEK films, respectively, using a two-component epoxy resin. The mechanical properties of the PEEK/Cu composites were characterized by the peel test sketched above. No improvement of the peel strength was observed by the mechanical pretreatment of the PEEK film, and the failure was due to a separation of the adhesive from the PEEK surface, as in the case of the lap test with the tensile bars described above that showed an improvement of adhesion by mechanically increasing the roughness.

Another coating process applied to the roughened PEEK films was physical vapor deposition of copper using a commercial DC magnetron sputtering device. The sample was located on a rotating disk and copper layers about 3 µm thick were generated. Adhesion properties of these thin layers were characterized by the forehead pull test. Surprisingly, the sputtered copper layer on the PEEK film blasted with glass beads showed a worse adhesion than on the untreated PEEK substrate, although a distinct roughness profile could be seen. This result may be explained by the formation of copper layers not completely covering all morphological features due to the effect of blasting. Thus, the overall pull strength may be weakened by local sites not coated by copper. The adhesive failure observed between the copper layer and PEEK substrate supports the assumption of weak interactions existing locally.

### 3.2. Corona Treatment

#### 3.2.1. Results from the Literature

Corona treatments have been widely applied to activate surfaces of items from polyolefins for the improvement of the adhesion of printing inks. Special equipment is available, and various techniques are state-of-the-art. The use of a commercial device to treat amorphous PEEK films in air, oxygen, argon, ammonia, or sulfur dioxide environments is reported in [20]. Surface properties were studied and the effect of the treatments on the lap shear strength of the films glued by an epoxide film adhesive to two steel adherends was measured. The surface roughness remained unaffected. The lap shear strength distinctly increased for the corona-treated samples, but for a discharge energy of 0.05 and 0.40 J/mm^2^ no difference was found. The failure mode changed from separation at the interface between PEEK film and adhesive for the untreated composite to cohesive failure within the adhesive or even the PEEK for the treated samples. Surprisingly, the increase in lap shear strength was very similar for all the five gases within the accuracy of the measurements. The mechanical performance conforms with the change of the O/C ratio of the surfaces characterized by XPS. A distinct increase of O/C was found after air and oxygen treatment, which, however, was only slightly higher than for the gases argon and ammonia that obviously should not contain any oxygen. This finding points to some limitations of the application of different gases in the device used, and the authors conclude that air may be sufficient to provide characteristic results for the corona treatment of PEEK. Of some interest are the results on films of various crystallinity. The lap shear strength without a treatment slightly decreased with crystallinity. The strength of the samples treated in air was distinctly higher than that of the untreated PEEK and remained unaffected by the degree of crystallinity. This result indicates the dominating role of the O/C ratio for adhesion due to the polarity of oxygen.

Similar results on the effect of corona treatment on adhesion properties have been reported for PEEK/carbon fiber composites in [21]. The dominating role of the PEEK matrix for adhesion properties was demonstrated.

#### 3.2.2. Studies on PEEK/Copper Laminates

For special applications, particularly in electronics, copper-coated PEEK films are of interest. Reports on developments in this field are not available from the literature. Therefore, our own studies were performed. For this purpose, 50 μm PEEK films and 18 μm copper foils were joined using an epoxy adhesive. These composites were mechanically characterized by a peeling test. The corona treatment was carried out in air with commercial laboratory equipment where the sample is fixed to a rotating plate. The roughness became smaller in comparison to the untreated sample. The peeling strength increased by a factor of four under the conditions chosen and the failure mode changed from adhesive failure of untreated PEEK to mixed appearances of fracture in the joined components. A similar change was observed in the forehead pull test.

The contact angle measured with purified water decreased by about 20° after corona treatment and indicated an enhancement of the surface energy of the PEEK substrate. This effect is due to a distinct increase of the O/C ratio found in the XPS spectra of PEEK, which effects a higher polarity at the surface.

Besides the lamination of copper foils, the sputtering process was applied for metallization. A first interesting result is the significant enhancement of the forehead pull strength of the untreated sample in comparison to the glued laminate. Starting from the distinctly larger value, a slight increase of the forehead pull strength by only 15% compared to the untreated PEEK film was observed for the layer sputtered on the corona-treated sample. Partial pullouts of the copper layer from the PEEK film were found to be going along with the failure of the untreated and treated samples. The polarity of the film being sputtered has a significant influence on the adhesion performance. If it is positive, the pull strength becomes about 25% lower than that of the untreated film.

In addition to films, the effect of a corona treatment on adhesion properties was studied with tensile bars by measuring the lap shear strength as described above. For these corona-treated samples, a surface layer was observed that could be easily removed by wiping. This layer consists of low-molecular-weight oxidized material (LMWOM). The cleaned glued parts showed a distinct increase in shear strength. A surface roughness did not occur, but the contact angle decreased, and the O/C ratio increased similar to the PEEK films, for which ablation residues were not observed. These results indicate the existence of activated surface layers underneath the LMWOM. For some applications, low-molar-mass degradation products have to be seen critically insofar as they may reduce the adhesion strength if not removed before the joining procedure.

### 3.3. Plasma Activation

#### 3.3.1. Results from the Literature

Plasma treatments and particularly those under low pressure have been in use for several decades. They are of special relevance for applications in the semiconductor industry. Due to the large energy of electrically charged particles and their high mobility, plasma has the ability to clean surfaces, but additionally it may change the constitution of surface molecules. Thus, plasma has been applied to polymeric materials to activate surface molecules and enhance properties such as adhesion, for example. A review of the plasma treatment of various polymers for the improvement of bonding is given in [22]. It represents an overview of the generation, effectiveness, and application of plasmas, but PEEK is not discussed.

The plasma etching of PEEK is compared to other treatments with respect to its efficiency on the lap shear strength of glued sheets in [16], and the corresponding surface characterizations are reported in [23]. The plasma treatment was performed in an oxygen atmosphere at a chamber pressure of 140 Pa and a power of 100 W for 15 and 30 min. The lap shear strength achieved was the highest compared to that of grit-blasted or chromium-sulfuric-acid-etched samples. From the XPS analysis, a significant increase of the O/C ratio followed in comparison to the untreated PEEK.

A very detailed analysis of the chemical groups generated by oxygen plasma was published in [24]. From XPS an O/C ratio of 0.17 was obtained for the methanol-rinsed PEEK as received and an O/C ratio of 0.42 was obtained after a plasma treatment at a power of 80 W and an oxygen pressure of 20 Pa for 3 min. After removing the ablation material by rinsing in methanol, an O/C ratio of 0.29 was found, which is distinctly higher than that of the initial sample and provides evidence for the additional incorporation of oxygen into the PEEK surface. A detailed analysis reveals a great variety of carbon/oxygen and carbon/hydroxyl groups such as phenols, carbonyls, carboxylates, and carbonates in the low-molar-mass surface layer, but significant concentrations of phenolic alcohols and acid groups with distinct dipole moments remain on the surface after rinsing. Furthermore, a signal corresponding to the shake-up mode is visible and is reduced compared to that in the initial PEEK. This result can be interpreted as a hint to the scission of some of the aromatic rings of PEEK by the plasma.

The effect of oxygen plasma treatment on adhesive bonding is reported for a PEEK/carbon fiber composite in [25]. An increase of lap shear strength and oxygen content was found, but no quantitative correlation could be established. Thus, the authors conclude that the oxygen concentration measured by XPS is not a useful parameter to predict the adhesion strength.

In another paper, PEEK/carbon-fiber composites were treated with oxygen, nitrogen, and argon plasmas, respectively. The adhesion was characterized by a pull test using special commercial equipment not further specified [26]. The adhesion strength increased by plasma treatment, but the data obtained were not very sensitive as the plasma parameters varied. Furthermore, the chemical composition of the different samples was not studied and, thus, no deeper insights into the activation mechanisms may be obtained.

PEEK/carbon-fiber composites treated with low-pressure plasma were compared with samples exposed to other treatments in [18]. Argon or oxygen plasmas were used at a microwave frequency of 2.45 GHz and a pressure of 20 Pa. The lap shear strengths of the two plasma-exposed samples were superior to those of the grit-blasted, corona-treated or chromium-sulfuric-acid-etched specimens. Surprisingly, the argon plasma resulted in a somewhat higher strength than the oxygen plasma. The XPS analysis did not offer a convincing explanation for this result and, thus, the authors concluded that the UV radiation of the plasma may be the decisive quantity for the chemical changes on the PEEK surface leading to an enhancement of adhesion.

The influence of different plasma gases on properties of 0.25 mm thick PEEK films glued to each other using an epoxide resin was studied in [27]. Oxygen, air, argon, and ammonia were used. The plasma treatments distinctly enhanced the adhesion strength, but at comparable treatment conditions the peel strengths and the lap shear strengths used for characterization were not significantly affected by the gas species. This result is supported by the XPS analysis that shows an increase of oxygen content by plasma treatment being nearly the same for oxygen, air, and argon and only somewhat lower for ammonia. However, this result raises questions regarding the source of oxygen in the cases of argon and ammonia that were not addressed by the authors.

Air plasma was applied to PEEK, and surfaces were characterized based on their dependence on treatment time [28]. Under the conditions chosen, a distinct decrease of the contact angle was found already after 25 s, which did not change much at longer times. Correspondingly, an increase of the oxygen content was observed by XPS. These results are in qualitative agreement with those of other studies. However, different are the findings on an increase of roughness with treatment time.

From the studies on plasma treatment discussed above, the general trend can be derived that this method may be used to improve the bonding properties of PEEK. However, some questions remain open, such as the efficiency of the different gases and studies that allow more detailed assessments of the effect of experimental conditions and the use of various adhesion partners and joining methods. Thus, our own studies are reported in the following sections.

#### 3.3.2. Detailed Studies on PEEK/Copper Laminates

PEEK films with a thickness of 50 µm were treated in a plasma chamber under oxygen and argon, respectively, at a pressure of about 100 Pa, a frequency of 13.56 MHz, and a power of 600 W. For example, the adhesion strength of the copper foil glued to the PEEK film by an epoxy resin was found to distinctly increase by several hundred percent in the peel test and the forehead pull test as well. Similar enhancements were found for argon plasma-pretreated composites.

However, in contrast to gluing, the sputtered copper layer showed only a slight increase of adhesion strength by about 20% in the forehead pull test after a pretreatment of the PEEK film in an oxygen plasma for 300 s. The longer treatment time of 900 s did not lead to any further enhancement. This increase of adhesion of the sputtered copper layer was much smaller than that found for the corresponding composites where a copper foil was glued to a PEEK film by an epoxy adhesive. It has to be mentioned, however, that the forehead pull strength of 0.82 N/mm^2^ for the copper-sputtered untreated PEEK was already distinctly higher than that of the glued copper foil of 0.16 N/mm^2^. The exposure to an argon plasma for 300 s led to adhesion properties similar to those for the untreated film. After 900 s, even a slight decrease was observed.

These results have to be discussed in light of the surface characterizations. For the oxygen-plasma-treated PEEK, the O/C ratio increased from 0.16 to 0.35 after 300 s and remained unchanged after 900 s. Within the accuracy of the measurement, the argon plasma treatment did not show a deviation of the oxygen content from that of the initial PEEK as measured by XPS. This result is in contrast to some findings in the literature (e.g., [18]), but expected for a pure argon plasma. At a first glance, the oxygen ratio unchanged by the argon plasma treatment is not in agreement with the contact angle measurements, however. After oxygen plasma treatment, the contact angle came down from 84° to around 25° due to the polarity of the oxygen groups incorporated. After the argon plasma exposure, about 50° were measured, while an unchanged angle would have been expected from the lack of oxygen uptake found by XPS. A difference in surface roughness was not observed for samples exposed to the various treatments and cannot be discussed as a reason for the different contact angles. Both plasmas led to a smoothening of the surface.

According to these analytical results, special effects of the plasma have to be discussed. Thus, the highly energetic plasma particles and the UV radiation found for plasma discharge may generate radicals or even crack molecules of the PEEK substrate. These moieties can increase the polarity of the surface, indicated by the decrease of the contact angle, and may support the bonding of the epoxy adhesive to the PEEK substrate. Their interactions seem to be less effective, however, than those between oxygen groups and epoxy molecules, because the failure strength after argon plasma treatment is lower than that with oxygen plasma. A distinct hint to changes of the molecular structure of PEEK by argon plasma treatment follows from the C 1s XPS spectra shown in [29]. For the untreated material, a shake-up satellite peak at 219.9 eV is found. This peak can be related to electron transitions in the aromatic ring, as described for the phenyl ring in polystyrene [30]. The peak for PEEK was not visible anymore after the treatment with argon plasma, indicating the disappearance of the aromatic ring. A similar observation on PEEK was reported in [31] where a decrease of the satellite peak after oxygen plasma treatment was related to a ring-opening reaction by cleavage of the aromatic structure.

### 3.4. Excimer-Laser Treatment

Another method for the surface treatment of polymers is laser irradiation. Laser techniques are very suitable insofar as devices with a wide scope of specifications are commercially available. In comparison to corona or plasma treatment, laser irradiation can be flexibly used because the laser beam may be directed and positioned by optical means. In principle, no special treatment chambers are necessary, as in the case of plasma, and the handling is environmentally friendly in comparison to blasting or chemical etching. Over the years, lasers have found a wide range of applications in medical and technical fields, and that is the reason why equipment with a large spectrum of specifications exists. Wavelengths from infrared to ultraviolet with various pulse energies and pulse lengths from femto- to nano-seconds are available. Particularly suitable for molecular interactions within polymeric materials are excimer lasers due to their high-irradiation energies and their short pulses that do not initiate thermal degradation but may lead to chain scissions and low-molar-mass layers on the surface of a substrate. This ablation has two effects. On the one hand, it can be used for the purification of surfaces, but on the other it may act as a boundary layer that weakens the adhesion required between two components.

Results from the literature and our own studies are reported in the following sections.

#### 3.4.1. Results from the Literature

**Figure 4:**
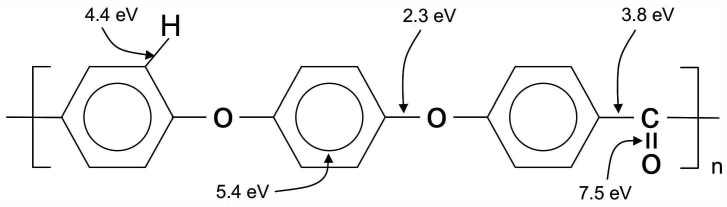
Bond energies of a PEEK molecule [32].

Preferentially, ArF and KrF lasers are used for the modification of PEEK. Their wavelengths of 193 nm and 248 nm, respectively, correspond to photon energies of 6.4 and 5 eV, which are in the range of various bond energies within the PEEK molecule, as can be seen from Figure 4.

Thus, a cleavage of bonds may occur by photochemical interactions, and new species can be generated by reactions with gas molecules from the environment. In [33], a cohesive failure of glued PEEK sheets pretreated with laser light of 193 nm is reported, but an adhesive failure is reported after irradiation with 248 nm. In both cases, the irradiation energies applied were below the ablation threshold. In the XPS spectra it was found that the overall O/C ratio increased with the number of pulses at 193 nm but decreased at 248 nm. The most pronounced differences occurred for the carboxyl group (C-C=O). It significantly increased for irradiation with the shorter wavelength but remained zero for the longer one. The higher content of polar oxygen groups may formally explain the better adhesion properties, but it is not understandable why the relatively small differences in photon energy can have such an effect since both are in the range of the bond energies within the PEEK. Similar results were published in [34]. The different reactions of the two wavelengths were further analyzed in [35], and an increase of carboxyl, peroxide and ester groups was found after an irradiation at 193 nm in an oxygen atmosphere. However, the conclusion that particular photochemically induced reactions may be related to excitations of the PEEK surface and the surrounding oxygen at 193 nm is not very elucidating.

The complexity of the reactions taking place during the irradiation of PEEK with an excimer laser becomes obvious from [36]. An ArF laser at 193 nm was used in an oxygen and argon atmosphere below the ablation threshold, and the change of adhesion of PEEK films glued to aluminum substrates was measured in a double cantilever beam test. For the samples irradiated in oxygen and argon as well, a distinct increase of failure energy was found, although the molecular analysis showed different results. As expected, the O/C content was higher in the oxygen atmosphere and increased with the number of pulses but remained unchanged under argon. This result is reflected by the polar component of the surface energy that became larger after the treatment in oxygen but was found to be the same in argon compared to the initial PEEK. A slight decrease of the shake-up mode, being comparable for oxygen and argon treatment, was observed. From the missing relationship between oxygen uptake and bonding efficiency in the case of argon, it was concluded that other effects in addition to the polarity of the surface come into play. Crosslinking at the PEEK surface under argon, which improves mechanical properties but weakens surface layers under oxygen, was postulated without specifying and proving the underlying reactions, however.

The effect of laser wavelengths larger than those of the excimer lasers above was studied in [37]. The aim of this work in the medical field was to modify PEEK with respect to surface roughness and wetting, which are seen as the main parameters affecting cell adhesion and other characteristics of implants. An Nd:YVO_4_ laser with wavelengths of 1064, 532 and 355 nm was used. The longest wavelength effected local burning spots, with the 532 nm radiation ablation was observed that produced small grooves. For both wavelengths, debris were found that resulted in poor adhesion and, thus, their use is not suitable for biomedical applications where good adhesion is required. The wavelength of 355 nm revealed a slight melting of the surface only, and a previous mechanically induced surface structure was preserved and nearly unchanged. The authors relate these appearances to the different absorption coefficients that increase with the wavelength. The contact angle characterizing the wetting behavior was found to be smaller at 355 nm than at the larger wavelengths. These results favor the treatment with the ultraviolet laser light for the improvement of the adhesion properties of PEEK. Studies on the chemical and physical reasons for this finding were not performed, but from the foregoing results reported above it can be assumed that the ultraviolet irradiation effected the scission of bonds within the PEEK molecules, which then became able to react with the oxygen from the surrounding air atmosphere, resulting in an enhancement of polar groups.

A contribution to the influence of laser wavelength on the modification of PEEK can be obtained from the results in [38]. The authors used a continuous CO_2_ laser with a wavelength of 10,600 nm, which is far in the infrared region. The irradiation doses were varied by drawing the samples at different velocities along the laser source. Surface roughness, contact angle and the chemical composition of the surfaces were measured after irradiation in air, argon and CO_2_ atmospheres. The roughness of the treated samples increased with decreasing feed velocity, corresponding to longer exposition times. The surface energies of the irradiated samples remained unchanged within the accuracy of the measurements, independently of exposition time and surrounding atmosphere. This result is reflected by the O/C ratio determined from XPS spectra that shows a slight enhancement only for the sample kept in air. However, a distinct decay of crystallinity was observed with exposition time that can be explained by the melting of surface layers under the infrared irradiation and a subsequent quenching faster than the crystallization rate of PEEK. Comparing the photon energy of the CO_2_ laser of around 0.1 eV with those of the bond energies of the PEEK molecule in Figure 4, the result of a chemical unchanged material is obvious.

These results from the literature demonstrate the importance of short wavelengths for the surface modification of PEEK. Thus, excimer lasers were used for special studies of surface modifications of PEEK with respect to improving its bonding properties to various copper coatings.

#### 3.4.2. Coating of PEEK Films with Copper

Of special interest, particularly with respect to applications in electronics, are copper-coated polymer substrates. Due to its high end-use temperatures and its inertness, PEEK has a special potential in this field. Thus, excimer-laser treatment was studied in detail by the authors for the adhesion of copper foils and thin copper layers on PEEK films. The copper foils were glued by an epoxy resin and the layers were produced by physical vapor deposition. The PEEK films of a thickness of 50 µm were irradiated in air by an XeCl excimer laser with a wavelength of 308 nm, corresponding to a photon energy of 4 eV. The pulse energy was 2 J and the typical pulse duration was 45 ns. Two energy densities of 0.6 and 3 mJ/mm^2^ were chosen. For both energy densities, ablation layers of low-molar-mass components were observed on the film surface, which were wiped off before the coating procedures. For the sample irradiated with the lower energy, a greyish color remained, indicating that degraded carbon-based residues may have been left. At the higher energy no ablation residuals could be visually noticed after wiping, which does not mean, however, that all degradation products were removed.

A change of roughness by the laser treatment could not be observed within the accuracy of the measurements. However, significant effects occurred for the contact angle of the distilled water used as the test medium. It increased from 84° for the untreated PEEK film to 146° for the irradiated samples nearly independently of the laser energy applied. This distinctly nonpolar behavior may be related to the carbon from the degradation process. After removing the layer, a contact angle of around 55° was measured for the two irradiated samples. The wettability distinctly enhanced in comparison to the untreated PEEK points to an increase in the polarity of the molecules of the surface layer by the irradiation. The reason may be seen in the generation of oxygen groups by bond scission, as discussed above for the corona- or plasma-treated samples.

However, surprisingly, an increase of O/C typical of corona or plasma treatment cannot be deduced from XPS spectra; rather, a slight decrease is found. This result may be due to carbon-rich degradation species not completely removed by mechanical wiping and, thus, overlaying the postulated oxygen increase as consequence of chain scissions by the laser light.

Very interesting are the results of the adhesion tests of epoxy glued copper foil and copper layers by PVD, respectively. The laser treatment with 0.6 J/mm^2^ enhanced the forehead pull strength of the glued foil by a factor of five, and the failure mode changed from adhesive failure to fracture within the substrate material. After irradiation with the larger laser energy of 3 J/mm^2^, only a marginal improvement of adhesion was observed, and the failure mode became adhesive again. Comparable results were obtained from the peeling tests.

The PVD coating showed different results. As mentioned before, the forehead pull strength of the copper layer on the untreated PEEK film was distinctly higher than that of the glued foil. Irradiation with 0.6 J/mm^2^ decreased the strength by about a factor of 2, whereas the mechanical behavior after 3 J/mm^2^ was not much different from that of the PVD-coated initial PEEK film. These results are difficult to understand without knowing the interactions of the sputtered Cu atoms with the ablation products and the chemical composition of the PEEK surface after wiping off the ablated species. Qualitatively it can be said, however, that carbon rich components remaining on the surface after laser treatment have a distinctly stronger effect on the physical vapor deposition of copper than on the processes underlying the gluing of the copper foil with the epoxy resin.

The improvement of the adhesion of the glued foil by an excimer-laser irradiation of 0.6 J/mm^2^, which is comparable to corona or plasma treatments, points to a negligible effect of the ablation layer. The reason may be that the debris still available after wiping are incorporated in the resin and, thus, the epoxy molecules can interact with the polar oxygen groups of the modified PEEK molecules. In the case of the higher laser energy, it seems feasible that some of the possibly larger amount of ablated material is left on the surface and disturbs the bonding reactions. Such an assumption would explain the only marginal increase of adhesion at the higher laser energy of 3 J/mm^2^.

Such an absorption of ablation debris is obviously not possible in the case of the copper layer from vapor deposition.

### 3.5. Etching

A relatively simple method for the surface modification of polymeric materials is etching with strong oxidizing acids. In practice, the application of such chemicals needs a lot of safety precautions, and their disposal is not environmentally friendly. Furthermore, a selective surface treatment with acids is difficult due to the use of dipping baths. Some studies can be found in the literature, which are shortly reviewed in the following sections.

#### 3.5.1. Results from the Literature

In [16], a significant increase of the lap shear strength of glued glass-fiber-reinforced PEEK is reported after etching with chromium sulfuric acid (CSA) composed of 75 g/L sodium dichromate in 300 g/L sulfuric acid. The adhesion improvement was comparable to that of the treatment with the oxygen plasma used. The kind of etching acid has a great influence on the effectiveness of surface modification, as was shown by the treatment with nitric-sulfuric acid, which did not exhibit any improvement of shear strength in comparison to the untreated material. In [23], studies on the surface composition of sulfuric acid-treated samples by XPS are published, which showed a distinct enhancement of the O/C ratio similar to those exposed to the oxygen plasma. In parallel, an increase of the polar component of the surface tension was observed. A detailed analysis of XPS spectra revealed that carbonyl oxygen accounted for most of the measured oxygen. The generation of carbonyl groups goes along with the main feature observed for hydrocarbons exposed to thermal or chemical oxidation conditions.

The effect of etching with chromic sulfuric acid on the lap shear strength of carbon-fiber-reinforced PEEK is compared with the efficiency of pretreatments by corona discharge or oxygen and argon plasmas [18]. Specific data on the composition of CSA and the etching treatment are not given, but it is documented that the shear strength obtained is comparable to that for the corona-treated sample. However, it was lower than after plasma treatment.

In [31], chemical etching and oxygen-plasma treatment of PEEK were compared with respect to biomedical applications. Potassium permanganate in phosphoric acid was used for etching. Adhesion studies are not shown, but physical and chemical characterizations are reported. The surface roughness after both treatments increased, and the enhancement was more distinct in the case of the plasma. The contact angle decreased significantly after plasma exposure and etching as well, but the decay was more pronounced for the plasma treatment. A similar ranking was found for the O/C ratio. It raised from 0.16 for the initial PEEK to 0.32 for the plasma-treated sample, whereas the value for the etched sample was just between these two. The authors concluded that the plasma and etching treatments chosen were powerful methods to obtain polar and hydrophilic PEEK surfaces that improve wettability and, thus, coating properties.

The adhesion improvement of copper to PEEK films using CSA etching is reported in [29]. The specific composition of the CSA is not given. The PEEK films were immersed at room temperature for 60 s, then rinsed with deionized water and dried in air. The peel force of a copper foil glued to the etched PEEK films was found to be significantly enhanced compared to the untreated film and was twice as high as that of copper foil on the sample pretreated by oxygen plasma.

In [39], etching of PEEK was studied to improve bonding to a cured acrylic resin for specific dental applications. Furthermore, 98% sulfuric acid was used in comparison to a pure abrasion treatment and a significant increase of bond strength was found in the specially designed mechanical test. Data on the chemical changes of PEEK by the acid treatment are not given.

These results from the literature show that the chemical etching of PEEK may be of some interest for surface modifications. Due to the different acids and etching procedures used, it seems to be difficult to derive general insights from the results published. Due to the lack of comprehensive analytical data, the chemical reactions between acids and PEEK are not known well enough, and the bonding mechanisms at the surface are not obvious. Thus, in the following, our own studies are reported, which were performed under defined conditions.

#### 3.5.2. Treatment of PEEK Films with Chromium Sulfuric Acid and Studies of Its Effect on the Adhesion of Copper

**Figure 5:**
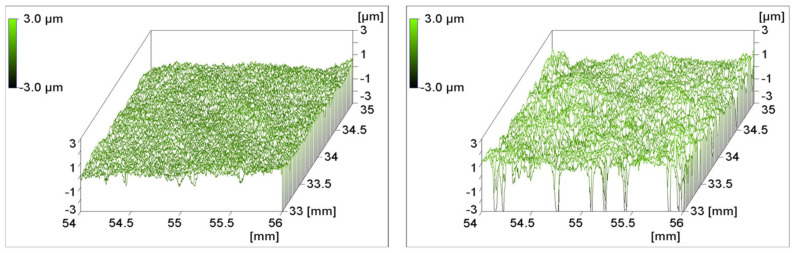
Surface roughness of a PEEK film dipped into chromium sulfuric acid for 5 s (**left**) and 60 s (**right**).

The chromium sulfuric acid used was prepared by mixing 80 mL sulfuric acid with 72 g potassium dichromate and 7 mL deionized water. PEEK films were dipped into this medium for 5 and 60 s, respectively. The surface roughness after etching is shown in Figure 5. After 5 s, the mean roughness already increased from 0.13 μm for the untreated film to 0.16 μm and the maximum roughness increased from 2.3 to 4.6 μm. After 60 s, the corresponding data were 0.52 and 14.6 μm. As seen in the right part of Figure 5, the acid treatment of 60 s severely attacked the PEEK film. Deep craters occur that hint to material abrasion, probably due to chain scissions preferably in the amorphous regions.

The contact angle decreased only marginally from 84.4° to 79.3° after a 60 s treatment with chromium sulfuric acid, whereas the O/C ratio increased from 0.16 to 0.26. Similar O/C ratios are reported in [5], but the contact angle decreased more distinctly. Chemical reactions effecting the obvious ablation of polymeric material from the surface and the generation of oxygen-containing moieties are proposed in [40]. The only little effect of the polar oxygen groups on the contact angle of the samples in Figure 5 can be explained by the more significant surface roughness observed, which may hinder the spreading of the test liquid due to capillary effects of the narrow and deep holes.

**Figure 6:**
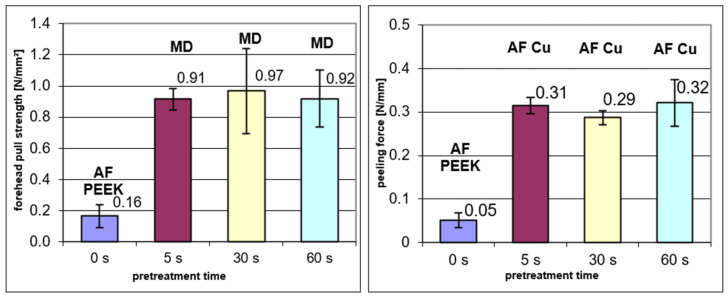
Pull strength (**left**) and peeling force (**right**) of glued copper foils after various pretreatment times of the PEEK films in chromium sulfuric acid. AF PEEK: Adhesion failure between PEEK film and adhesive, AF Cu: Adhesion failure between copper foil and adhesive, MD: Disruption of PEEK film below glued copper foil.

As in the case of the surface treatments by corona, plasma or laser irradiation discussed above, the acid-treated PEEK films were metallized either by the gluing of a copper foil or physical vapor deposition. The results on the glued composites are shown in Figure 6. The forehead pull strength increased significantly after a dipping time of only 5 s. The longer times of 30 and 60 s did not further change the pull strength within the accuracy of the measurements. The peel strength in the right diagram shows the same behavior: a distinct enhancement after 5 s, which is independent of dipping times up to 60 s. Obviously, the failure behavior changed with the treatment. For the untreated PEEK films, a failure between the film and the adhesive is observed; after dipping in chromic sulfuric acid the PEEK film with the glued copper foil is disrupted when the pull test is applied, and in the peel test the bond between copper foil and adhesive fails. These results indicate a modification of the PEEK surface and an improvement of the bonding properties with respect to the epoxy adhesive by the acid treatment.

A different picture is obtained for physical vapor deposition (PVD). The forehead pull strength of the copper layer on the untreated PEEK film—that is higher by a factor of five in comparison to the copper foil glued to the untreated film—does not show any further improvement by the treatment with chromium sulfuric acid. There was an improvement in adhesion for the glued copper foil by etching, but the negligible effect for the PVD coating is in line with the effect of the other pretreatments reported above.

## 4. Discussion

In Table 1, the most essential features of the various treatments with respect to the surface modification of PEEK and the resulting bonding properties to copper are summarized.

Moreover, data on the two metallization processes of gluing copper foils by epoxy adhesives or of physical vapor deposition of copper layers are compared. Considering the untreated sample, it is surprising that the forehead pull strength of the sputtered layer is about five times higher than that of the glued foil. Electrostatic interactions between the copper cations of the PVD process and the polar ether and keto groups of PEEK may be the approach to an explanation. Such binding forces do not exist for the electrically neutral epoxy adhesive.

The treatments described do show different effects on surface properties and bonding. As expected, and well-known from the literature, sandblasting significantly increases the surface roughness but leaves the chemical composition characterized by the O/C ratio unchanged. Within the uncertainty of the measurements, the contact angle was found to remain the same. This finding is in agreement with the assumption that sandblasting effects a mechanical abrasion, but no breakage of chemical bonds within the PEEK occurs. The forehead pull strength of the glued copper foil is slightly enhanced, but the peel strength is the same as for the untreated sample. From these results it has to be concluded that the mechanical roughening of the PEEK film has only a small influence on the adhesion behavior of the glued copper foil. Surprisingly, the forehead pull strength of the thin copper layer obtained by PVD is significantly decreased in comparison to the untreated sample. This may be due to the fact that the maximum roughness of the blasted PEEK film of about 8 μm is distinctly higher than the 3 μm average thickness of the deposited copper layer and, thus, the metallization is assumed to be not uniform, giving rise to local regions uncovered by copper and subsequent weak adhesion.

The effects of corona treatment, with decreasing surface roughness and contact angle and enhanced O/C ratio, are in agreement with reports from the literature. The improvement of adhesion of the glued copper foil measured by the two methods described above is obvious and can be attributed to the polarity of the created oxygen groups. Somewhat surprising is the pull strength of the PVD copper coating, which is only slightly higher than for the untreated sample. This may be due to the limited significance of the test method at large adhesion strengths, as discussed above. The observation of various failure modes occurring within one test sample supports the uncertainty of the test.

Oxygen plasma smoothens the surface of the PEEK foil and distinctly enhances the O/C ratio. In parallel, the contact angle becomes significantly lower, and the characteristic adhesion strengths of the glued copper foil determined with the two methods increase by a factor of six in comparison to the untreated film. Again, oxygen intake and adhesion improvement conform with each other. Similar to the corona treatment, the adhesion of the sputtered copper layer characterized by the forehead pull strength is only weakly enhanced.

Interesting are the results that argon plasma and excimer-laser irradiation, respectively, lower the contact angle but do not show an oxygen increase. This result reflects the inert argon atmosphere during the plasma treatment and the thin surface layer of degraded material by laser irradiation as addessed in Section 3.4.2. Nevertheless, the characteristic mechanical adhesion data for the glued copper foil are distinctly improved. As discussed above, a cleavage of the aromatic rings of PEEK can be postulated according to the XPS spectra, and the epoxy resin may be able to react with the radicals created. In the case of the laser treatment, the adhesion of the sputtered copper layer is distinctly lower than that of the untreated sample. Carbonic decomposition products on the surface postulated from its visually observed light discoloration may act as a weak boundary layer and hamper the effective interaction between copper cations and PEEK molecules addressed above.

The chromium sulfuric acid (CSA) treatment attacks the PEEK film in two ways. The roughness is distinctly enhanced, and the chemical reactions of the strong oxidizing acid with PEEK leads to high O/C ratios. As can be seen from Table 1, the characteristic mechanical adhesion data reach the best marks, although the contact angle is only weakly affected. The small change of the contact angle may be due to the strong fissures from the CSA attack that hinder the spreading of the test liquid. Considering the very small effect of the roughness increase by sandblasting on the adhesion of the glued copper foil, the oxidizing effect of CSA has to be seen as decisive for the adhesion improvement.

## 5. Conclusions

In agreement with the literature, it was found that PEEK surfaces can be modified in different ways. However, the treatments applied to one substrate material and the comparable characterizations performed in this work provide data from which the optimal suitability of a process for special applications can be assessed. The following results concerning surface treatments of PEEK films have been obtained.

Sandblasting increases the roughness of PEEK but leaves the chemical composition and the surface angle unchanged. Its technical application is not very challenging.

Corona treatment decreases the sample roughness. Preferentially used in an ambient environment, the O/C ratio and consequently the polarity of PEEK molecules is enhanced and, thus, the contact angle, which is related to the spreading of a liquid on a surface, becomes smaller. Thus, commercially available corona equipment is a convenient tool for surface modifications of PEEK.

Similar results can be obtained by the use of oxygen plasma, but the technique is more complicated to handle.

The application of argon plasma offers surface modifications and an improvement of copper foil adhesion in cases where an oxygen incorporation is not desirable. It smoothens the PEEK surface and decreases the contact angle but leaves the oxygen content of the surface layer unchanged. The experimental effort necessary is high, however.

Excimer laser treatment can be used for reducing the surface roughness and the contact angle without oxygen uptake. Using corresponding optical devices for positioning the light beam, local surface modifications can be carried out, but the technical realization of this method is rather elaborate. Furthermore, a carbon layer may be observed at the surface that disturbs the bonding of coatings.

Etching with acidic liquids, and with chromium sulfuric acid in particular, is very effective with respect to enhancing the O/C ratio at the surface. In parallel, the surface roughness is significantly increased, however, and that may be of disadvantage for applications where smooth surfaces are required. Furthermore, dipping is the preferential treatment mode and, thus, locally selective activations are difficult to realize.

In addition to the comparison of the surface modifications obtained with well-defined methods for a typical commercial PEEK, the effect of different treatments on bonding properties was assessed using the same materials, coating processes, and mechanical tests.

As a representative example related to applications, a copper foil was glued to the treated PEEK film with a commercial epoxy adhesive. Peel strength and forehead pull strength were used to mechanically characterize the bonding behavior. Increasing the roughness is not sufficient to achieve a significant improvement of bonding. Rather, special treatments with corona, plasma or laser irradiation are necessary to effect molecular changes within the PEEK that provide binding reactions with the epoxy resin. However, quantitative relationships between the mechanical characteristics and contact angle or O/C ratio could not be found. As a rule of thumb, it can be said, however, that a higher O/C ratio leads to a better adhesion of the epoxy glue. Additionally, a low O/C ratio does not definitely mean bad adhesion. As the results for the argon and laser treatments in Table 1 show, a decrease in contact angle and good adhesion of the glued copper foil can be obtained although the O/C ratio is close to that of the untreated PEEK. These results are discussed in light of the molecular changes of the PEEK by UV irradiation, typical of argon plasma and excimer-laser treatment.

As an epoxy glue is the bonding partner of PEEK, it is expected that the results obtained on the PEEK/copper laminate can also be transferred to other composites based on PEEK films using the same type of adhesive. For other base materials and adhesives, however, particular tests are necessary due to the decisive role of chemical interactions.

The limitations of mechanical tests for the general assessment of surface pretreatments on bonding properties become obvious from the results on the thin copper layers by physical vapor deposition in Table 1. Due to the high bonding efficiency between the untreated PEEK film and sputtered copper, the forehead pull strength to be used is only able to distinguish between treatments that lead to a decrease of bonding in comparison to the untreated sample.

The studies presented show the complexity of the surface modification of PEEK and demonstrate how difficult it is to draw general conclusions desirable for a time- and cost-efficient product development. Nevertheless, the results provide a base for some preselections to start with in the case of surface modifications of PEEK for various applications.

## Figures and Tables

**Table 1:** Effect of various treatments on surface properties of a PEEK film and its adhesion to copper.

Treatment	Untreated	Blasting	Corona	Plasma		XeCl-Laser	CSA
				Oxygen	Argon		
**R_a_ [µm]**	0.13	0.40	0.08	0.10	0.08	0.08	0.52
**R_max_ [µm]**	2.3	8.4	2.5	1.5	2.0	2.7	14.6
**Contact angle [°]**	84.4 ± 1.2	79.2 ± 4.4	58.8 ± 2.8	28.2 ± 2.8	47.5 ± 3.0	53.7 ± 3.7	80.4 ± 1.1
**O/C**	0.16	0.16	0.22	0.35	0.15	0.14	0.27
**σ_E_ [N/mm²]**	0.16 ± 0.07	0.25 ± 0.12	0.84 ± 0.16	1.00 ± 0.08	0.65 ± 0.05	0.81 ± 0.14	0.97 ± 0.27
**F_E_ [N/mm]**	0.05 ± 0.02	0.05 ± 0.04	0.22 ± 0.03	0.33 ± 0.03	0.35 ± 0.07	0.24 ± 0.07	0.32 ± 0.05
**σ_PVD_ [N/mm²]**	0.82 ± 0.09	0.31 ± 0.11	0.94 ± 0.13	1.00 ± 0.14	0.77 ± 0.19	0.47 ± 0.06	0.90 ± 0.10

Contact angle: Measured in distilled water, R_a_: Mean roughness, R_max_: Maximum roughness, CSA: Chromic sulfuric acid; σ_E_: Forehead pull strength of epoxy glued copper foil, F_E_: Peel strength of epoxy glued copper foil; σ_PVD_: Forehead pull strength of copper layer by physical vapor deposition (PVD).

## Data Availability

Not applicable.
